# Graphene Reinforced Composites as Protective Coatings for Oil and Gas Pipelines

**DOI:** 10.3390/nano8121005

**Published:** 2018-12-04

**Authors:** Xingyu Wang, Xiaoning Qi, Zhibin Lin, Dante Battocchi

**Affiliations:** 1 Department of Civil and Environmental Engineering, North Dakota State University, Fargo, ND 58018, USA; xingyu.wang@ndsu.edu; 2 Department of Coatings and Polymeric Materials, North Dakota State University, Fargo, ND 58018, USA; Xiaoning.qi@ndsu.edu (X.Q.); Dante.Battocchi@ndsu.edu (D.B.)

**Keywords:** nano-modified high-performance coating, dispersion methods, graphene nanoplatelets, corrosion mitigation, nanocomposite, gas and oil pipelines

## Abstract

Corrosion and corrosion-induced damage have resulted mostly in malfunctions and sometimes even in failures of metallic structures, including oil and gas pipelines. In this study, new high-performance composite coatings were developed by incorporating nanoparticles in the polymer resins with applications to oil and gas pipelines. The graphene nanoplatelets under different concentrations were used to prepare the epoxy-based nanocomposites and were then evaluated through mechanical and electrical tests. The integration of high-speed disk and ultrasonication were adopted as the dispersion technique to overcome nanoparticle agglomeration. Electron microscopy techniques were used to investigate the agglomeration. The new composites were qualitatively and quantitatively evaluated in terms of contact angle, surface roughness, adhesion to the substrate, corrosion resistance, and abrasion resistance. The results suggested that the composite with 0.5~1.0 wt.% of the graphene nanofillers led to the largest improvement in both mechanical and electrochemical properties. Distribution of nanoparticles in the matrix was observed using scanning electron microscopy and surface roughness using atomic force microscopy. Large agglomeration that was observed at the higher concentrations mainly resulted in the reduction of corrosion resistance and abrasion resistance.

## 1. Introduction

The United States consists of over 2.6 million miles of gas and oil pipelines. As the major pipeline system, the onshore transmission metallic pipelines for gas and hazardous liquids demand a high standard of safety. Corrosion is one of the leading causes of failures of metallic pipelines in the United States and worldwide [1,2].

The report [3] revealed that corrosion of onshore gas and liquid transmission pipelines were responsible for a cost of over $7 billion. One reason for this is that most of the oil and gas pipelines are made from low-carbon steel, meaning it is vulnerable to corrosion as the main element is iron [4,5]. Despite there being various protective coating systems that are often used as strategies for corrosion control, there are still challenges to mitigate corrosion of the metal [6,7,8,9].

Polymeric coating is widely used to provide a protective film for the metal that can prevent the direct contact between the substrate and the corrosive environment [4]. The aggressive environments that pipelines are usually exposed to, such as severe abrasion, hydrothermal aging, and chemical degradation, however, degrade these protective coatings and decrease their capability of corrosion control. For instance, epoxy coating is often used as a corrosion protection film for metallic structures due to good corrosion resistance (in the short run), good adhesion to substrates, and relatively low cost [10,11]. However, the neat epoxy coating often experiences durability issues, including decomposition/blistering/delamination, which is mainly due to the fact that micro-pores that are generated during the curing process of the neat epoxy coating allow electrolytes to penetrate through the film and reach the coating-metal interface, thereby leading to a significant reduction in the barrier performance in the long term [11,12]. In recent years, many researchers have been attempting to incorporate nanoparticles in polymer matrices to develop high-performance coatings [4,13,14,15,16,17,18,19,20,21,22,23,24,25,26,27,28,29]. The use of nanomaterials as reinforcement in polymers, such as using carbon nanotubes (CNTs), has been studied since the 1990s [13,14,15]. These nanoparticles reinforced polymers have displayed enhanced mechanical, thermal, or electrical conductivity properties. Different to one-dimensional line-shaped CNTs, two-dimensional graphene nanoplatelets have been reported as being used for the modification of polymeric coatings with considerable improvements of mechanical and electrochemical properties [15,30,31,32,33,34,35].

Graphene, a two-dimensional carbon nanomaterial with the building block of natural graphite, is currently the strongest known material [18]. It is a planar monolayer of carbon atoms with the C=C bond length of 0.142 nm [36]. Previous studies have shown that graphene nanoplatelets (GNPs) have many extraordinary electrical, mechanical, thermal, and optical properties, including high electron mobility (250,000 cm^2^/V_s_ at room temperature), thermal conductivity (5000 W m^−1^ K^−1^), and Young’s modulus of 1 TPa [19,37,38,39]. Moreover, some derivatives of graphene, such as graphene oxide and reduced graphene oxide, can also serve as effective barriers to ionic transport and abrasive-resistance enhanced additives in coatings.

Much research has been conducted on the implementation of graphene for multifunctional coatings [10,11,12,13,14,15,16,17,18,19,20,21,22,23,24]. Liu et al. [20] fabricated graphene/epoxy coatings with two different contents, 0.5 and 1.0 wt.%. Their results showed that the presence of GNPs enhanced the corrosion resistance of epoxy coating and the coating with 0.5 wt.% graphene exhibited better performance. Chang et al. [21] discussed the impacts of the dispersion of graphene on the coating performance. Liu et al. [22] investigated the tribological and anticorrosion properties of epoxy coating reinforced by graphene nanoparticles. They observed that dramatic material degradation occurred when the content of graphene was higher than 0.5 wt.% due to the agglomeration of the nanoparticles. Different to previous work [22], Yu et al. [23] found that a higher content of graphene nanoparticles still provided high performance in resistance to corrosion and gas barrier. They evaluated the nanocomposites with varied contents of graphene from 0.5 to 2.0 wt.% and concluded that 2.0 wt.% of GNP additions achieved a great improvement of anti-corrosion performance and gas barrier properties. Monetta et al. [24] experimentally evaluated the performance of graphene/epoxy coating with 1.0 wt.% of graphene nanoparticles. Their results indicated that graphene improved the anticorrosion properties of polymers. They also pointed out that the curing process of epoxy was not affected by the addition of graphene and that no significant changes were found in the adhesion and water repellency of the cured coating. To sum up, graphene shows promising solutions for coating systems. Due to different focuses in each study, some conclusions may conflict with each other. For instance, it is still debatable how much graphene should be used in order to obtain the stronger reinforcement for the nanocomposite coatings. Also, although there are studies on the GNP or derivatives of graphene, most of the efforts were focused on either electrical, mechanical, or tribological properties of the nanocomposites [20,21,23,25,26,27,28,29], and only a few studies [21,22,24] provided other mechanical performances of graphene/polymer as well as corrosion resistance, as is often required in metallic structures.

Therefore, the objectives of this study aim to evaluate the effect of incorporating GNPs into epoxy adhesive as additions to develop high-performance coatings for oil and gas pipeline applications. High-speed disk and ultrasonication were introduced in the dispersion process. Both electrical and mechanical properties were evaluated with various concentrations of nanocarbon additives. The effects of incorporating nanofillers into polymer matrix were experimentally evaluated by the following tests: (i) electrochemical impedance spectroscopy (EIS) test, (ii) Taber abraser test, (iii) pull-off strength test, and (iv) water contact angle test. In addition, we also evaluated nanofiller reinforcement in the polymer matrix by using scanning electron microscopy (SEM) and surface roughness using atomic force microscopy.

## 2. Experimental Program

This section describes the experimental methods to characterize the effectiveness of incorporating nanomaterials into the coating where the epoxy is selected as a prime as a representative. The detailed information of the materials, dispersion methods, and the procedure of the experiments are described below.

### 2.1. Material

Graphene nanoplatelets without any modification were selected from commercially-available products (Cheap Tubes Inc., Cambridgeport, VT, USA) where the average specific surface areas of the graphene are 500–700 m^2^/g with an average thickness of 8–12 nm. EPON™ Resin 828 resin and Epikure 3175 (Hexion Inc., Columbus, OH, USA) were used as the baseline prime matrix to mix with the nanoparticles. EPON™ Resin 828 is a bisphenol A/epichlorohydrin derived liquid epoxy resin and can obtain good mechanical, adhesive, dielectric, and chemical resistance properties when crosslinked with appropriate curing agents. EPIIKURE™ Curing Agent 3175 (Hexion Inc., Columbus, OH, USA) was used as the curing agent of EPON™ Resin 828. Q-panel steel panels (Q-Lab Corporation, Cleveland, OH, USA) with surface roughness of 0.5~1.14 μm were used as substrates for the prepared coatings.

### 2.2. Fabrication of Nanofiller-reinforced Epoxy Composites and Test Sample Preparation

**Figure 1:**
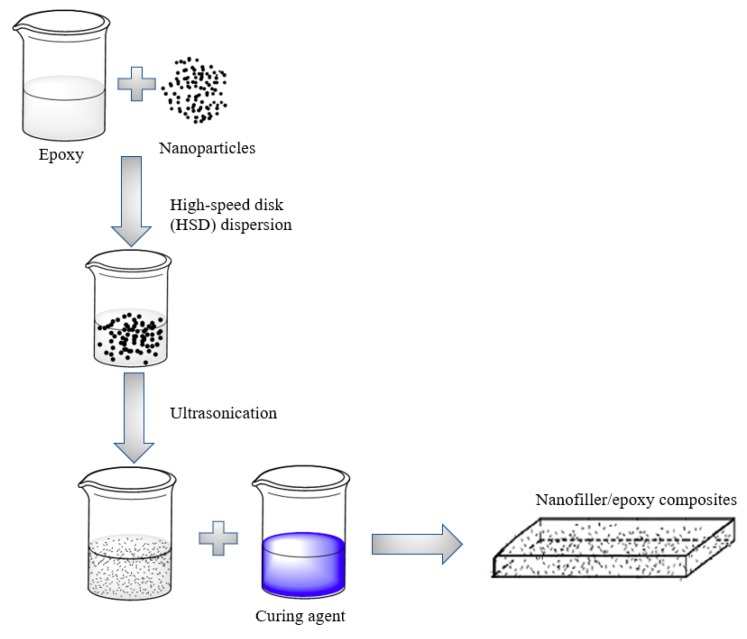
Schematic of the fabrication process of the nano-modified composite coating.

As schematically illustrated in Figure 1, the nanofiller composite coatings were synethesized using various nanofiller concentrations from 0.1 to 3.0 wt.%. Both High-speed disk (HSD) and ultrasonication dispersion methods were herein integrated to enhance the dispersion and alignment of the nanoparticles in the epoxy matrix. HSD dispersers (high-speed impellers) with a rotation speed of 4000 rpm was first used for 30 min to break down the particles by providing shear stress during the high-speed rotation. After the HSD process shown in Figure 1, the nanofiller particles solution were added into the EPON 828 and were subjected to the multrasonication (Misonix S1805 sonicator using a 30 s on/off cycle with a 19-mm probe at 100% amplitude) with a total duration of 60 min to ensure the proper dispersion of the graphene solution in the polymer matrix. Following up the dispersion, the resin was mixed with the EPIIKURE™ Curing Agent 3175 (with a 1:1 mole ratio) by mechanical stirring for another 10 min.

As such, the new developed nanofiller composite coatings were then coated on the standard Q-panel steel substrates with a dimension of 76 × 152 × 0.8 mm, and three samples per group were used for the characterization, as presented below. The panels were used without any modification and were cleaned with acetone before the coatings were applied. Single layer coatings were applied and cured at room temperature for 24 h. All the coated samples were allowed to completely dry for a few days before testing. Elcometer 415 thickness gauge were used to measure the thickness of dried coating films, and an average thickness of 110 ± 5 μm were obtained for the tested samples. For simplicity, the test samples were labeled in terms of graphene contents, unless stated otherwise. For instance, 0.5%G-Epoxy denotes the composite reinforced with 0.5 wt.% graphene, while 3.0%G-Epoxy is the composite with 3.0 wt.% graphene. The neat epoxy samples were used as the controlling reference.

### 2.3. Characterization Methods

#### 2.3.1. Corrosion Resistance of the Composite Coating Using EIS

The EIS is often used to characterize the electrochemical behavior of a coating. The EIS tests of the coated samples were herein conducted using the Gamry Reference 600 potentiostat/Galvanostat/ZRA (Gamry Instruments Inc., Warminster, PA, USA) with counter and reference electrodes that were immersed in electrolyte solution and were then evaluated based on the interfacial properties between the substrate and conductive electrolyte solution. Specifically, a 30-mm diameter O-ring glass tube with a silicon joint was clamped on the test panels. A saturated calomel electrode reference electrode was used for the reference electrode; while a platinum mesh was employed as the counter electrode and the test panel was worked as the working electrode. The glass tube was filled with 1.0% NaCl solution at all times during the test. The test was carried out by applying both DC potential and small superimposed AC excitation to a working electrode which was immersed in the conductive solution. After AC and the potential measurements were collected over a very wide range of frequency excitation, the collected data of current and potential of the electrochemical cell were converted into the impedance vs. frequency curve. The impedance-frequency plot analysis produced useful information about the coating and dielectric properties.

Moreover, the ASTM standard B117-Salt Spray Test using Q-Fog CCT chamber (Q-Lab Corporation, Cleveland, OH, USA) at NDSU were conducted as an accelerated durability test of the test samples for 200 h to evaluate their overall long-term performance.

#### 2.3.2. Abrasion Resistance of the Composite Coating Using the Taber Abraser Method

Abrasion resistance of a coating is usually evaluated by either the Falling abrasive method (ASTM D 968) or the Taber abraser method (ASTM D 4060). In this study, the Taber abraser method was selected due to the fact that such a method provides a better representation of the wear resistance on a wide range of materials by selecting different abrading wheels and applied loads. Each specimen was loaded for a total of 1000 cycles under an applied load of 1000 g in accordance with ASTM D 4060 and the rotational speed of 72 rpm with two CS-10 abrading wheels. With such an applied load and the rotation, the abrading wheel generated a circular abrasion mark with an approximate area of 30 cm^2^. The abrasion resistance was then calculated through the mass loss in a specified number of abrasion cycles with the selected loads.

#### 2.3.3. Adhesion of the Composite Coating Using Tensile Button Testing

Adhesive bonds of the coating to its metal substrate were characterized by tensile button testing. Dollies were glued to each specimen, and the pull-off strength was measured during the experiment. Scotch weld 460 (3M, Co., Maplewood, MN, USA) was used to glue the dollies, and the test area was abraded with sandpaper (100 grit or finer) to enhance the bonding strength between the dollies and the tested coatings. The test was performed 24 h after the dollies were applied. The test area was isolated with other parts of the coating by die cutting. An adhesion tester was used to apply tension load normal to the test surface. The adhesion strength was measured when the dolly was detached by the tension load.

#### 2.3.4. Contact Angle Testing of the Composite Coating

A contact angle test of the composite was conducted to understand the surface modification when adding nanoparticles, based on the ASTM D7334. Images were captured by the camera and were processed by a computer to calculate the angle between the water droplet and the contact surface.

#### 2.3.5. Microstructures Using Scanning Electron Microscopy and Atomic Force Microscopy

**Figure 2:**
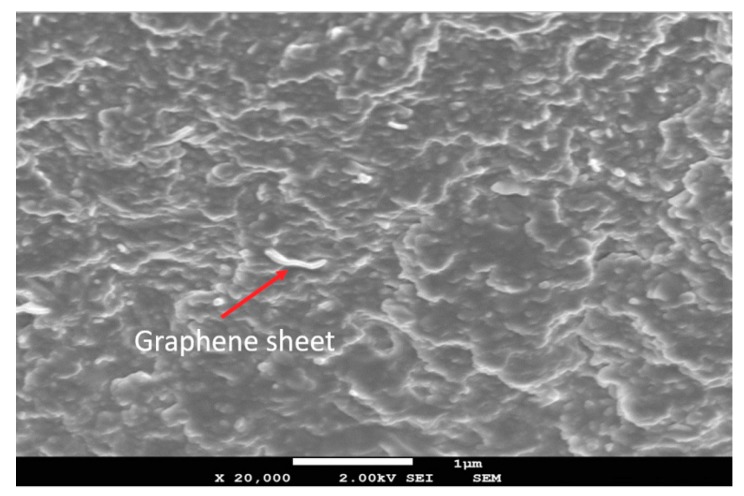
SEM (scanning electron microscopy) micrograph of a graphene sheet in epoxy matrix (scale bar = 1 µm).

Besides that, the scanning electron microscopy (SEM) technique was used to observe the nanoparticles and their distribution in the epoxy matrix. The SEM was carried out by a field emission scanning electron microscope (FE-SEM), with a JSM-7600F Schottky (JEOL USA, Inc., Peabody, MA, USA) at 2 KV. Note that 2-D graphene alone is optically active and, thus, can be easily discriminated by microscopes. However, after being dispersed in a polymeric matrix, the discrimination of graphene sheets in the microstructure is a challenging job, as identified by other researchers [40,41,42]. As illustrated in Figure 2, one graphene sheet was identified embedded in the composite matrix when adjusting the optical density. Differently, agglomerates of nanoparticles that have slightly large size are easier to be classified and, for this reason, the SEM herein tends to identify potential agglomerates of the nanoparticles experienced in the matrix, as discussed in Section 3.1.

Moreover, surface roughness analysis was performed with an atomic force microscopy, a Nanoscope IIIa system. By scanning the surface with a sample-probe, the height changes of the tested samples were recorded to generate three-dimensional images that were used to determine the surface roughness (*R_a_*).

## 3. Results and Discussions

### 3.1. Corrosion Resistance of the New Nanocomposites in the Short Term

**Figure 3:**
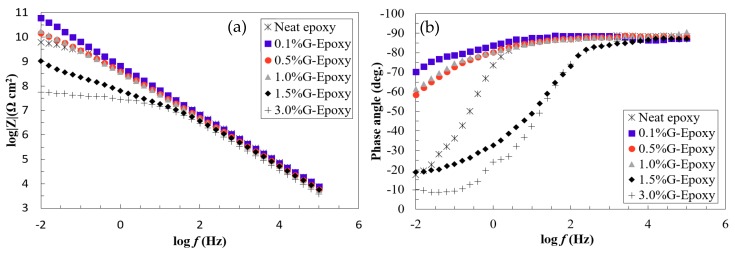
Bode plots in terms of (**a**) impedance and (**b**) phase angle for the test samples.

The effect of graphene nanoparticles on the electrochemical behavior of the new composite coating was evaluated by the EIS technique using single sinusoidal excitation of 10 mV magnitude under the frequency domain ranging from 100 kHz to 0.005 Hz at stabilized open circuit potential. EIS diagrams of the coated steel samples in 3.5 wt.% NaCl were illustrated for the immediate immersion as Bode plots, as shown in Figure 3a,b.

As illustrated in Figure 3a,b, the neat epoxy group exhibited a clear bend in the low frequency region of the Bode plot where its phase angle was 17° at a lower frequency, suggesting that the electrolyte penetrated the coating film to contact with the metal substrate and the corrosion reaction occurred, as confirmed elsewhere [43]. Different to the neat epoxy, the phase-angles for the test samples, 0.1%G-Epoxy, 0.5%G-Epoxy, and 1.0%G-Epoxy, reached up to 60° and mostly approached 90° in a wide frequency region shown in Figure 3a, with a straight line in the impendence curve in the Bode plot. This observation was consistent with the previous findings [11,44], indicating that the new nano-modified coatings offered the enhanced resistance to corrosion.

**Figure 4:**
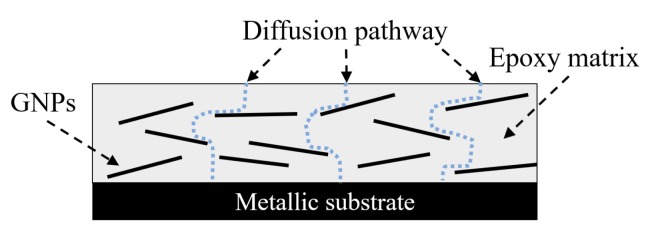
Mechanism of nanoparticles for enhanced corrosion resistance.

The mechanism of the reinforced effects by the incorporation of graphene in terms of enhanced corrosion resistance was schematically illustrated in Figure 4. The improvements of corrosion resistance properties of the composites mainly result from: (a) the GNPs are impermeable materials that form excellent barriers against the corrosive solutions by dramatically reducing electrolyte pathways and increasing the diffusion length for oxygen and water; and (b) the well dispersed nanoparticles fill the pores in the organic matrix and, thus, lead to a reduced porosity for the coatings, as similarly observed by other researchers [12,22].

**Figure 5:**
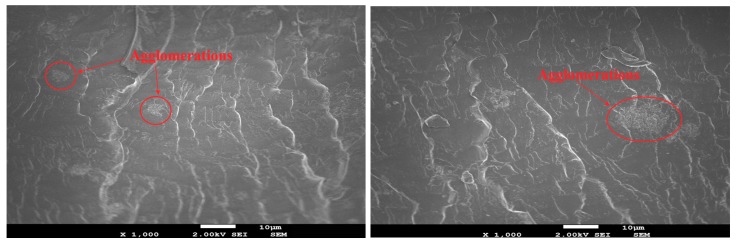
Agglomeration observed in the (**a**) 1.0 and (**b**) 3.0 wt.% composites (scale bar = 10 µm).

With the increase of graphene contents, the groups 1.5%G-Epoxy and 3.0%G-Epoxy, however, led to a different trend. Rather than strengthening the polymer, these samples exhibited a reduction in performance in terms of smaller phase angles, as shown in Figure 3a,b, and correspondingly weaker resistance to corrosion, as compared to other groups with lower graphene contents or even the neat epoxy. The results could be partially explained due to particle agglomerates when under high volume of graphene nanoparticles, even using the integrated dispersion methods as presented in this study. Some studies have shown that the agglomerates of nanofillers were not able to fill the micro-pores of the polymer matrix, however also lead to the formation of defects, thereby causing a decrease in the performance of the nanocomposites [45,46,47]. Cross-sectional microstructures of the composites with 1.0 wt.% (1.0%G-Epoxy) and 3.0 wt.% (3.0%G-Epoxy) graphene contents were demonstrated in Figure 5a,b. Due to strong van der Waals forces between nanoparticles, slightly small agglomerates, with a size of around 1.0 μm (see Figure 5a), were observed in some regions of the matrix in the group 1.0%G-Epoxy. A severer and larger agglomeration area was observed in higher GNPs contents, such as 3.0%G-Epoxy shown in Figure 5b where the agglomerated particle was around 15 μm. These results were accordingly consistent with the measured EIS data that the higher graphene contents led to reduced resistance to corrosion.

**Figure 6:**
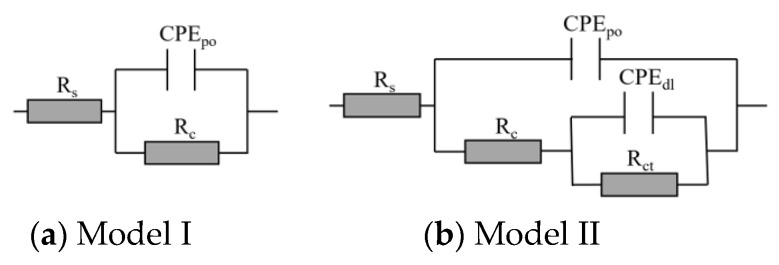
Equivalent electrical circuit models used for the EIS (electrochemical impedance spectroscopy) data (**a**–**b**).

In addition, two equivalent electrical circuit models, illustrated in Figure 6, were employed to fit the EIS data for Nyquist plots to further describe the electrochemical behavior of the composites under different stages. The AC impedance plots in accordance with the test EIS data reveal the relation of the coating capacitance and the coating resistance for the metallic substrate [2,48,49]. Model I in Figure 6a describes an intact coating that is capable of providing excellent barrier properties against environmental attacks. Model II is used for describing the degradation of coatings. The model parameters of these models are: *R*_s_ is the solution resistance, *R*_c_ is the coating resistance, *R*_ct_ is the charge transfer resistance, *CPE*_dl_ is the constant phase element of the double-charge layer, and *CPE*_po_ is the constant-phase element of the coating [11].

**Figure 7:**
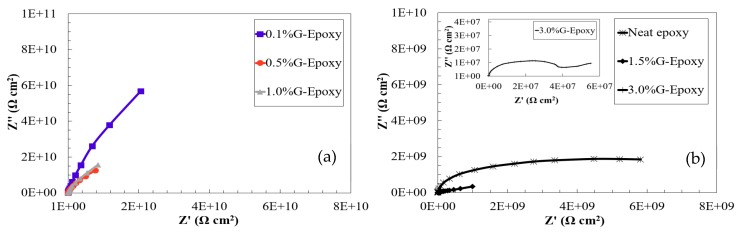
Nyquist plots for: (**a**) 0.1~1.0%G-Epoxy and (**b**) 1.5~3.0%G-Epoxy and the reference.

The impendence plots shown in Figure 7a,b exhibit good agreement to confirming the positive effects of adding graphene nanoparticles into the polymer. As illustrated in Figure 7a, the EIS data of the test samples, Groups 0.1%G-Epoxy to 1.0%G-Epoxy, fitted well with the model I shown in Figure 6a, suggesting that the presence of graphene nanoparticles in terms of an effective barrier for the substrate (see schematics shown in Figure 4), as well as potentially good dispersion of the nanoparticles, enhanced the barrier performance of the composite coatings and, thus, exhibited excellent corrosion resistance, consistent with the findings in Bode plots in Figure 3.

Differently, the neat epoxy samples and others with higher graphene contents (i.e., 1.5%G-Epoxy and 3.0%G-Epoxy) had a semi-circuit curve in Nyquist, as presented in the second model shown in Figure 7b. At this stage, model II was found to better predict data for neat epoxy, 1.5%G-Epoxy, and 3.0%G-Epoxy samples, and it appeared that there were diffusion paths for electrolyte to reach the coating-substrate interface and initiated coating degradation. Particularly, the group 3.0%G-Epoxy, illustrated in the inserted plot in Figure 7b, exhibited the semi-circuit curve in the Nyquist plot and the lowest impedance values in the low frequency region of the Bode plot. As a result, 3.0%G-Epoxy had the weakest corrosion resistance and cannot provide an effective corrosion protection as a coating.

### 3.2. Corrosion Resistance of the New Nanocomposites in the Long Run

**Figure 8:**
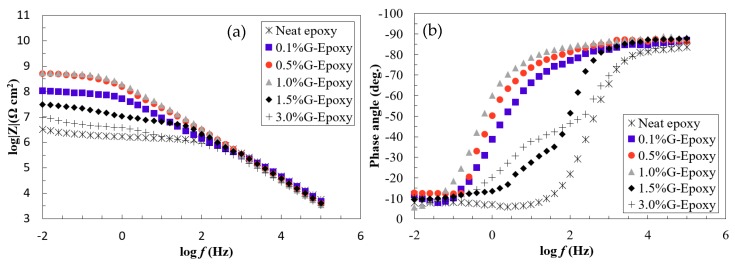
Bode plots of (**a**) impedance and (**b**) phase angle for the test samples after 200 h exposure.

**Figure 9:**
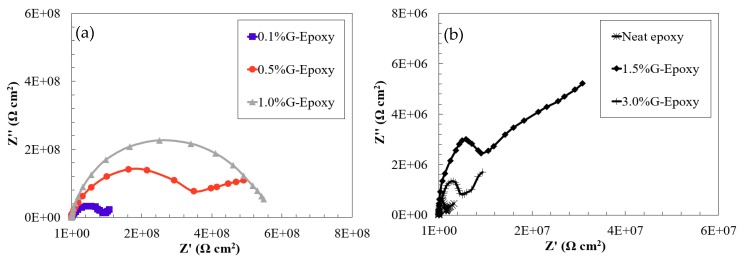
Nyquist plots for the test samples after 200 h exposure: (**a**) 0.1–1.0%G-Epoxy and (**b**) 1.5–3.0%G-Epoxy and the reference.

Long-term performance of the coatings was evaluated using accelerated durability salt spray tests. The test results after the samples had been exposed to salt spray for 200 h are illustrated in Figure 8 and Figure 9 by the Bode and Nyquist plots. Clearly, the neat epoxy coating displayed the most degradation in the barrier property after accelerated durability tests and had the most inferior long-term performance. As illustrated in Figure 8, the impedance modulus at 0.01 Hz of the neat epoxy decreased about two and a half orders, and a second semi-circle was observed in the Nyquist plot as well. This observation confirmed that the corrosion resistance decreased during the long-term exposure, where the NaCl solution penetrated the neat epoxy films and initialized the interface degradation. Thus, the minimum region of the phase angle plot shifted toward high frequency and confirmed that the corrosion protection of the neat epoxy was damaged during the exposure, similar to the observation by another researcher [20].

As clearly illustrated in Figure 8 and Figure 9, the durability tests further confirmed that using graphene nanoplatelets as nanofillers to modify the polymeric coatings significantly enhanced their long-term barrier performance. Among all the GNP/epoxy coatings, the group 1.0%G-Epoxy exhibited the best performance in terms of significant improvements of durability. The 1.0%G-Epoxy had a slight decrease in the impendence modulus at 0.01 Hz and displayed one capacitive loop in the Nyquist plot, as shown in Figure 9a, suggesting that the coating film with 1.0% GNP content still provided an effective barrier for the substrate after 200 h exposure. Similar to the short performance, the higher graphene contents had little contribution to the corrosion resistance mainly due to the graphene agglomeration. Both Figure 8 and Figure 9 showed that the groups, 1.5%G-Epoxy and 3.0%G-Epoxy, exhibited the inferior long-term performance, which was identical to the neat epoxy ones.

### 3.3. Abrasion Resistance of Nano-Reinforced Composites

**Figure 10:**
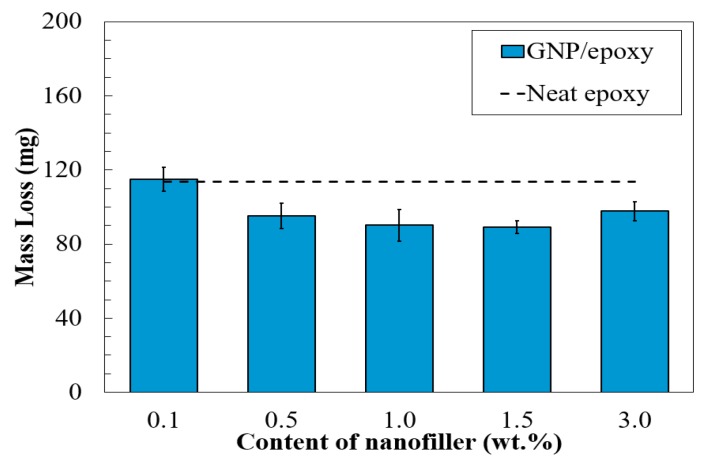
Mass loss of nanofiller/epoxy composites.

The abrasion resistance of a coating is the ability against the removal of materials from its surface by rubbing, scraping, or erosion actions that the real-world coatings often experience and, thus, low abrasion resistance of a coating could lead to low operational durability [50]. The results of the abrasion test for the samples in terms of mass loss were statistically evaluated by the analysis of variance with a confidence level of 99% and were presented by bar graphs in Figure 10, where the mass loss of the neat epoxy in dashed lines was selected as a reference for comparison.

As clearly illustrated in Figure 10, the group 0.1%G-Epoxy had a mass loss of 110 mg, which was identical to that of the neat epoxy, suggesting that the addition of 0.1 wt.% graphene nanoparticles resulted in no increase to the abrasion resistance of the coating. With the increase of graphene contents (>0.1 wt.%), the test samples exhibited enhanced abrasion resistance by 20% reduction in mass loss. Particularly, the groups 1.0%G-Epoxy and 1.5%G-Epoxy displayed the maximum enhancement in abrasion resistance.

Similar to the previous observation in corrosion resistance, the group 3.0%G-Epoxy had no further increase in abrasion resistance (see Figure 10) when adding more graphene particles, mainly due to the agglomeration at the higher local concentration.

### 3.4. Adhesive Bonding Strength of Nano-Reinforced Composites to the Substrate

**Figure 11:**
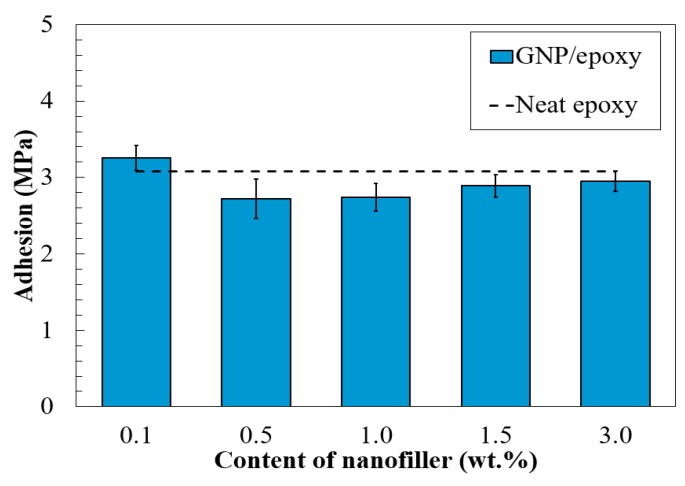
Adhesion strength of the composite coatings to the substrate.

Adhesion between the coating and substrate is another important factor to contribute to the barrier properties for the coating system [21]. The adhesion strength of the developed composite coatings with various graphene contents was herein measured in accordance with the ASTM D4541, and their results are plotted in Figure 11, where the results of the neat epoxy, about 3.0 MPa, are shown with dashed lines for comparison.

As illustrated in Figure 11, group 0.1%G-Epoxy maintained a high adhesion strength of 3.2 MPa, while other test samples, such as 1.0%G-Epoxy and 3.0%G-Epoxy, were 2.75 and 2.95 MPa, respectively. Clearly, the samples with the higher graphene contents exhibited some levels of reduction, however they still stayed within 10%, as compared to the case of the neat epoxy, suggesting that there were no significant changes of the adhesion.

### 3.5. Surface Roughness of the Composite Coating

**Figure 12:**
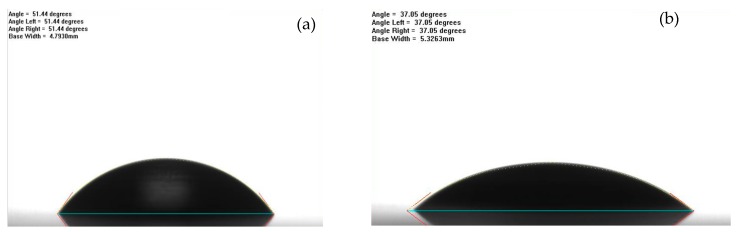
Water contact angle for (**a**) the neat epoxy, (**b**) group 1.0%G-Epoxy.

**Figure 13:**
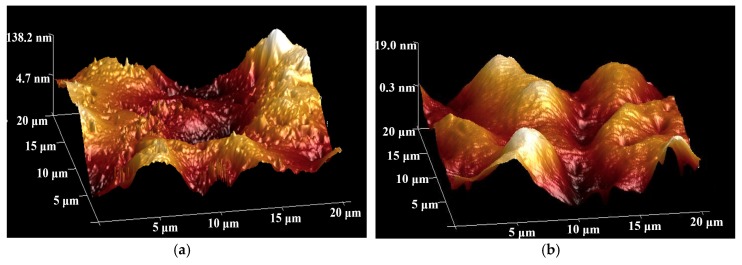
AFM images of (**a**) the neat epoxy, (**b**) group 1.0%G-Epoxy.

Figure 12 was plotted to present the water contact angle of the test samples. The contact angle of water for the neat epoxy was about 55 degrees, while the composite coatings with different graphene contents exhibited a reduction in the contact angle, with an averaged value of 41 degrees, as listed in Table 1, indicating that incorporating graphene slightly decreased the hydrophobicity of the surface, which was further confirmed by the surface roughness, *R_a_*, of the nanocomposites by atomic force microscopy, as shown in Figure 13. Clearly, the surface profile of the epoxy resin samples (see Figure 13a) was visually much rougher than those of the composites with the addition of the nanoparticles (see Figure 13b). The averaged *R_a_* value of the epoxy resin was 29 nm, while the graphene-based composites were only less than 5 nm. The results demonstrated that there was a significant decrease in the surface roughness by adding a small amount of nanofillers.

## 4. Conclusions

The incorporation of graphene nano-platelets into the polymer matrix were evaluated for their potentials as high-performance coatings. The factors affecting the composite coatings, including nanoparticle contents, and corresponding electrochemical, abrasion resistance, and adhesion properties, were investigated with specific conclusions, as shown below:

(a) Graphene-based composite coatings exhibited enhanced mechanical and electrochemical properties that enabled the accommodation of the needs in wide structural applications, while maintaining high adhesion strength to the substrate.
(b) Electrochemical behaviors of the test samples revealed that the nanocomposites with 0.1 to 1.0 wt.% of GNPs offered effective barrier properties for corrosion mitigation in both short- and long-term performances. Due to the agglomeration when increasing the high content of nanoparticles, the samples with 1.5 or 3.0 wt.% of GNPs exhibited some levels of reduction in corrosion resistance.
(c) A similar trend was observed in the abrasion resistance. The results demonstrated no significant influence on the water contact angle and adhesion test by incorporating graphene in the epoxy resin. The graphene-based composite coatings with 0.5 to 1.5 wt.% of GNPs provided an over 20% increase in abrasion resistance in terms of low mass loss, as compared to the neat epoxy.
(d) Results of the adhesion strength of the composites to the substrate showed that incorporation of the nanoparticles has minor effects on their interfacial bonding, showing a reduction within 10%. Moreover, the contact angle tests and surface roughness of the composite coatings both supported that the graphene particles smoothened the surface texture, as compared to the neat epoxy samples.
(e) The composite coatings that were reinforced by graphene nanofillers display promising results in terms of enhanced mechanical and electrical properties, thereby offering the potential for widespread real-world applications, including oil and gas pipelines and bridges. Note that conclusions were mainly drawn from the findings in a short-term manner. Although the accelerated durability tests by 200 h exposure of salt spray confirmed the enhanced performance of the graphene-loaded composite coatings over conventional neat epoxy, it still necessitates the further investigation of the long-term durability tests in future studies.

## Figures and Tables

**Table 1:** Surface roughness and contact angle of the composite coatings.

Items	Group	
	Neat Epoxy	GNP (Graphene Nanoplatelets)/Epoxy
Surface Roughness (*R_a_*, nm)	29 ± 5	4 ± 2
Contact Angle (degrees)	55 ± 10	41 ± 7
